# Surface Modification of Wood Flour via ARGET ATRP and Its Application as Filler in Thermoplastics

**DOI:** 10.3390/polym10040354

**Published:** 2018-03-22

**Authors:** Martin Kaßel, Julia Gerke, Adrian Ley, Philipp Vana

**Affiliations:** Institute of Physical Chemistry, University of Göttingen, Tammannstr. 6, D-37077 Göttingen, Germany; mkassel@gwdg.de (M.K.); julia.gerke@stud.uni-goettingen.de (J.G.); adrian.deinert@stud.uni-goettingen.de (A.L.)

**Keywords:** ARGET ATRP, wood flour, composite, surface modification, graft polymerization, mechanical properties, tensile testing, solvent casting, poly(methyl acrylate) (PMA)

## Abstract

Wood flour is particularly suitable as a filler in thermoplastics because it is environmentally friendly, readily available, and offers a high strength-to-density ratio. To overcome the insufficient interfacial adhesion between hydrophilic wood and a hydrophobic matrix, a thermoplastic polymer was grafted from wood flour via surface-initiated activators regenerated by electron transfer-atom transfer radical polymerization (SI-ARGET ATRP). Wood particles were modified with an ATRP initiator and subsequently grafted with methyl acrylate for different polymerization times in the absence of a sacrificial initiator. The successful grafting of poly(methyl acrylate) (PMA) was demonstrated using attenuated total reflection Fourier transform infrared (ATR-FTIR) spectroscopy, scanning electron microscopy (SEM), thermogravimetric analysis (TGA), and water contact angle (WCA) measurements. To confirm the control over the polymerization, a cleavable ATRP initiator was immobilized on the particles, allowing the detachment of the grafted polymer under mild conditions. The grafted particles were incorporated into a PMA matrix using solvent casting and their influence on the mechanical properties (Young’s modulus, yield strength, and toughness) of the composite was investigated. Tensile testing showed that the mechanical properties improved with increasing polymerization time and increasing ratio of incorporated grafted particles.

## 1. Introduction

Composites are made by combining two or more components to give a new material with enhanced properties. One early example of composites is the utilization of mud bricks reinforced with twigs or straw as a construction material [1]. The idea of composites was not invented by mankind; nature already provided high-performance composites in the form of wood. Wood has a highly sophisticated cellular structure which consists of rigid cellulose fibers embedded in a soft matrix of lignin and hemicellulose [2,3]. Wood is a particularly suitable filler for composites because it is cheap, renewable, very abundant on earth, and offers a high strength-to-density ratio. Over the last decades there has been a rapid growth in interest in wood-reinforced thermoplastics due to their outstanding properties such as high durability, light weight, and low maintenance [4]. Additionally, wood and polymer wastes can be used in these products, which will help to close the loop for conserving natural resources (cradle-to-cradle concept) [5]. However, wood has several disadvantages such as poor wettability, dimensional stability (shrinking and swelling behavior), and susceptibility to microorganisms [1,6]. Furthermore, the hydrophilic nature of wood, which originates from the high amount of surface hydroxyl groups, leads to poor compatibility in a hydrophobic thermoplastic matrix and, therefore, to low tensile strength and durability [2,7]. Graft polymerization is a powerful tool for surface modification to achieve better adhesion to the matrix. Graft polymerization can be done through treatment of the wood surface with aqueous solutions of hydrogen peroxide with ferrous iron or ceric ammonium nitrate, resulting in the formation of radicals on the surface. Afterwards, the resulting wood surface is immersed in a suitable monomer solution (compatible with the intended polymer matrix) for a certain period of time [8]. However, the radicals are formed under harsh conditions which result in a partial destruction of the surface. A more elegant way is the utilization of surface-initiated reversible-deactivation radical polymerization (RDRP) methods, since they can be conducted under mild conditions and the properties of the polymer can be tailored [9,10]. Atom transfer radical polymerization (ATRP) is one of the most commonly applied reversible-deactivation radical polymerization techniques. Particularly, the broad range of commercially available monomers, simple experimental setup, and readily available initiators and catalysts have contributed to its popularity in both industrial and academic science [11,12]. Surface-initiated ATRP (SI-ATRP) is a well-suited technique for the modification of surfaces with polymers, due to the fact that a vast amount of surface-tethered functional groups can be converted into ATRP initiators in a straightforward fashion with commercially available substances [12,13,14]. In particular, natural substances offer numerous superficial hydroxyl groups, which can be used for graft polymerizations. SI-ATRP has been successfully conducted on silk [15]; chitosan [16]; rice husk [17]; lignin [18,19]; cellulose [20,21,22,23,24,25,26,27]; and lignocellulosic material, such as jute fibers [28], wood pulp [29,30], and wood in form of flour and fibers [31,32,33,34]. Furthermore, SI-ATRP can be conducted exclusively on the surface without the presence of an initiator in solution. Therefore, the formation of an untethered polymer, which otherwise has to be removed after the polymerization, is prevented [35]. Besides these advantages, ATRP has two decisive drawbacks. Firstly, the polymerization must be conducted under a completely inert atmosphere because oxygen acts as a radical trap. Secondly, the required amount of transition metal, most commonly Cu(I), can be problematic with regards to toxicology and economics [13]. In 2006, a novel, more robust, and environmentally friendly ATRP technique called activators regenerated by electron transfer (ARGET) ATRP was introduced [36,37]. ARGET ATRP relies on slow and steady in situ regeneration of the Cu(I) from Cu(II) by a reducing agent throughout the polymerization. Due to the constant Cu(I) generation and large excess of reducing agent, ARGET ATRP can be performed with a significant reduction of copper and under limited amounts of oxygen.

The aim of this study was to modify the surface of wood flour used as a reinforcing filler material in a hydrophobic polymer matrix. To the best of our knowledge, there are no studies on the modification of wood flour via surface-initiated ARGET ATRP with respect to further use as a filler material in thermoplastics. Utilization of SI-ARGET ATRP allowed us to graft a hydrophobic polymer on the wood surface to improve the compatibility between wood and the polymer matrix. Both the amount of surface-tethered polymer as well as the amount of grafted particles incorporated into the matrix were investigated with regard to their influence on the mechanical properties of the composite. 

## 2. Materials and Methods

### 2.1. Materials

Acetone (ACS reagent, VWR Chemicals, Radnor, PA, USA); anisole (99%, Acros Organics, Geel, Belgium); ascorbic acid (AsAc, reagent grade, Sigma Aldrich, Saint Louis, MO, USA); azobisisobutyronitrile (AIBN, ≥98%, Fluka, Buchs, Switzerland), which was recrystallized from methanol; 2-bromoisobutyryl bromide (BIBB, 98%, Sigma Aldrich); Copper(II) bromide (CuBr_2_, 99%, Sigma Aldrich); 2-cyano-2-propyl dodecyl trithiocarbonate (98%, Sigma Aldrich); dichloromethane (DCM, HPLC Grade, Fluka); 4-dimethylaminopyridine (DMAP, 99%, Sigma Aldrich); dithiothreitol (DTT, Cleland’s reagent, ≥99%, Sigma Aldrich); 2-hydroxyethyl disulfide (technical grade, Sigma Aldrich); methanol (ACS reagent, VWR Chemicals); methyl acrylate (MA, 99%, Acros Organics), which was passed through a column containing aluminum oxide (basic, Brockmann I, 150 mesh, Sigma Aldrich) before use and stored at 4 °C until use; oxalyl chloride (98%, Alfa Aesar, Heverville, MA, USA); propylene glycol methyl ether acetate (PGMEA, ≥99.5%, ReagentPlus^®^, Sigma Aldrich); *N*, *N*′, *N*′, *N*″, *N*″-pentamethyldiethylenetriamine (PMDETA, 99%, Sigma Aldrich); 2-propanol (IPA, ≥99.5%, Sigma Aldrich); pyridine (Py, SeccoSolv, Merck KGaA, Darmstadt, Germany); succinic anhydride (≥99%, Sigma Aldrich); tetrahydrofuran (THF, ACS reagent, VWR Chemicals); toluene (Tol, ACS reagent, VWR Chemicals); triethylamine (TEA, ≥99%, Sigma Aldrich); wood flour (Arbocel^®^, natural raw cellulose, Grade C 100, J. Rettenmaier & Söhne GmbH & Co. KG, Rosenberg, Germany, particle size was 70–150 μm with spruce as dominant component).

### 2.2. Pretreatment of Wood Flour

Wood flour was extracted using a Soxhlet apparatus using dichloromethane for 16 h, followed by drying at 60 °C under reduced pressure. Finally, the flour was ground with a mortar and pestle and kept in a desiccator until use.

### 2.3. Immobilization of the ATRP Initiators

The immobilization of the initiator was inspired by Schwellenbach et al. [38]. One gram of dry wood flour was suspended in a solution of 100 mL DCM (90 wt %) containing triethylamine (TEA, 5 wt %). The resulting suspension was cooled to 0 °C and 2-bromoisobutyryl bromide (BIBB, 5 wt %) was added dropwise over 15 min and stirred for additional 30 min. The reaction mixture was stirred at room temperature for 23 h. Afterwards, the flour was filtered off and washed with copious amounts of tetrahydrofuran, acetone, 2-propanol, and dichloromethane; dried at 60 °C under reduced pressure; and stored in a desiccator until use. The immobilization of the disulfide initiator was conducted in a similar fashion, but in a solution of DCM (93 wt %) containing pyridine (2 wt %) and initiator (5 wt %).

### 2.4. Synthesis of Poly(Methyl Acrylate) (PMA) as Polymer Matrix

Methyl acrylate (MA, 5.3 mL, 58.1 mmol, 480 eq.), 2-cyano-2-propyl trithiocarbonate (40 mg, 0.166 mg, 1.0 eq.), and azobisisobutyronitrile (AIBN, 2.0 mg, 12.0 μmol, 0.1 eq.) were dissolved in toluene (10.5 mL) and degassed with argon for 10 min at 0 °C. The mixture was polymerized at 60 °C for 23 h, cooled down to room temperature, precipitated twice in methanol, and dried at 80 °C under reduced pressure. The molecular weight of poly(methyl acrylate) (PMA) was 3.6 × 10^4^ g·mol^−1^, the dispersity was 1.1, and it had a glass transition temperature of 19 °C (measured via DSC).

### 2.5. Polymerization on Wood Surfaces via ARGET ATRP

The initiator-modified wood particles (200 mg) were suspended in a solution of methyl acrylate (10 g, 116 mmol, 400 eq.), anisole (10 g, 93 mmol), CuBr_2_ (6.5 mg, 29 μmol, 0.1 eq.), and PMDETA (50 mg, 290 μmol, 1.0 eq.) before ascorbic acid (AsAc, 51 mg, 290 μmol, 1.0 eq.) was added. The flask was sealed and degassed with argon for 5 min. Afterwards, the reaction mixture was heated to 60 °C for the specific period (0.75 to 9.5 h). When the polymerization time was reached, the particles were filtered off, washed with tetrahydrofuran and acetone, dried at 60 °C under reduced pressure, and stored in a desiccator.

### 2.6. Synthesis and Cleavage of the Disulfide Initiator 

The synthesis of the ATRP initiator containing a disulfide bond was synthesized according to literature, except that the acid chloride was freshly prepared before immobilization. The cleavage of the disulfide-modified grafted wood (100 mg) was conducted using DTT (160 mg) and TEA (0.3 mL) in THF (10 mL). Afterwards, the wood particles were washed with an excess of THF and dried [39]. 

### 2.7. Preparation of Composite Materials

Poly(methyl acrylate) (PMA, 4 g, see Section 2.4) was dissolved in propylene glycol methyl ether acetate (PGMEA, 8 mL) to obtain a highly viscous solution. Wood particles (0–10 wt %, see Section 2.5) were added to this solution and the mixture was stirred until the particles were well dispersed (4 to 6 h). Dogbone-shaped specimens were prepared via solvent casting of the mixture by slowly drying over several days at 100 °C under reduced pressure.

### 2.8. Methods

#### 2.8.1. Size-Exclusion Chromatography (SEC)

Molar mass distributions were determined with an Agilent 1260 infinity system (Agilent, Santa Clara, CA, USA) consisting of an autosampler (Agilent 1260 ALS G1329B), an isocratic HPLC pump (Agilent 1260 Infinity ISO), a PSS-SDV (Polymer Standard Services) precolumn, three PSS-SDV separation columns (8 × 300 mm, particle size 10 µm, pore sizes of 10^6^, 10^5^, and 10^3^ Å) and an RI detector (Agilent 1260 G1362A). The setup was maintained at 35 °C and tetrahydrofuran (THF) was used as the eluent (flow rate of 1 mL·min^−1^). The system was calibrated via a conventional calibration method using narrowly PMMA standards (PSS, *M*_P_ = 800 to 1,600,000 g·mol^−1^) and toluene as internal standard. The molar masses of PMA were obtained using the Mark–Houwink parameters (*K* = 1.95 × 10^−2^ mL·g^−1^, *a* = 0.660) according to the principle of universal calibration [40,41]. All polymer samples (4 mg·mL^−1^) were filtered prior to injection (VWR, PTFE filter, 0.45 µm). 

#### 2.8.2. Attenuated Total Reflectance Fourier-Transform Infrared Spectroscopy (ATR-FTIR)

ATR-FTIR spectra were recorded using a Bruker IFS 88 (Bruker Optik GmbH, Ettlingen, Germany), equipped with a Harrick MVP 2 Star^TM^ ATR unit, a KBr beam splitter, a MCT detector, a globar, and a tungsten halogen lamp. The spectra were recorded with 32 scans per sample, in a range from 750 to 4000 cm^−1^, and at a resolution of 2 cm^−1^. 

#### 2.8.3. Scanning Electron Microscopy (SEM) and Energy-Dispersive X-ray Spectroscopy (EDX)

Scanning electron microscopy was recorded on an FEI Quanta FEG 250 (FEI Company, Hillsboro, OR, USA) equipped with an Everhart-Thornley detector. Prior to investigation, the samples were coated with gold. The images were taken using an acceleration voltage of 30 kV in high vacuum with a magnification of 2000. Energy-dispersive X-ray spectroscopy measurements were conducted three times per sample using the same acceleration voltage but recorded with a SSD (Silicon Drift Detector, EDAX Inc., Mahwah, NJ, USA). Bromine content was given as weight percent (wt %). Errors were given as the error of the respective integral.

#### 2.8.4. Differential Scanning Calorimetry (DSC)

DSC measurements were performed using a NETZSCH DSC 214 Polyma (Netzsch, Selb, Germany) with an automatically controlled liquid nitrogen cooling device in a temperature range from −50 to 150 °C. Measurements were performed with a heating rate of 10 °C·min^−1^ and a constant nitrogen flow of 40 mL·min^−1^.

#### 2.8.5. Tensile Testing

Tensile testing was conducted on a ZWICK & ROELL Z2.5 instrument (Zwick ROELL, Ulm, Germany) equipped with an air conditioner at 24.9 ± 0.3 °C. All measurements were performed with a strain rate of 0.25 mm·s^−1^ and a loading speed of 25 mm·min^−1^. The cross section of the specimen was taken at three different places and averaged. The contact pressure was 0.8 bar. Tensile data (Young’s modulus *E*, toughness *U*_T_, and yield strength σ_y_) reported herein were averages taken from at least three specimens per sample. The Young’s modulus (*E*) was calculated in the linear region of 0.05% to 0.20% elongation. Errors were given as maximum error. The data were collected and analyzed with the computer program testXpert II (Zwick ROELL) or ORIGINPRO 8.5G (Originlab, Northampton, MA, USA).

#### 2.8.6. Thermogravimetric Analysis (TGA)

Thermogravimetric analysis was performed with a NETZSCH TG 209 F3 Tarsus instrument (Netzsch, Selb, Germany). The sample was placed in an aluminum oxide crucible and heated from room temperature to 1000 °C with a heating rate of 10 °C·min^−1^ and under a constant nitrogen flow of 20 mL·min^−1^. The data were collected and analyzed with the computer program NETZSCH Proteus Thermal Analysis 6.1.0 (Netzsch).

#### 2.8.7. Water Contact Angle (WCA) Measurements

Water contact angle measurements were conducted using the static sessile drop technique on a Dataphysics OCA 15EC (Dataphysics Instruments GmbH, Filderstadt, Germany) with a measuring range from 0 to 180°, equipped with an LED backlight and a VGA camera with 752 × 480 pixels. Measurements were conducted at 21 °C and with droplets of 5 μL demineralized water. Prior to WCA measurements, the wood flour was dried at 60 °C under reduced pressure and pressed into a pellet with a diameter of 1 cm. 

#### 2.8.8. Fluorescence Microscopy

Dried wood flour containing thiol groups was immersed in a solution of 32 μg·mL^−1^ fluorescence dye (Abberior LIVE 510) in PBS (phosphate-buffered saline, pH 7) and shaken overnight in the dark. Afterwards, the flour was washed four times with PBS, centrifuged, and put on a microscope glass slide (Thermo Fisher, Waltham, MA, USA). Fluorescence images were taken with a microscope Leica DMi8 (Leica Microsystems GmbH, Wetzlar, Germany) equipped with a 63× oil immersion objective. The excitation was performed with an argon krypton laser, which was set to a gain of 500 mV and an intensity of 2%. The excitation wavelength was set to 488 nm and the detection range set to 500–570 nm. 

## 3. Results and Discussion

### 3.1. Polymerization of Wood Surfaces via ARGET ATRP

**Scheme 1:**
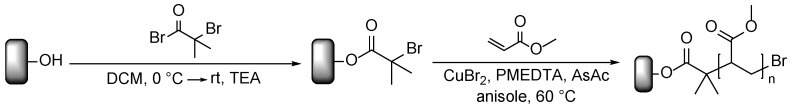
Initiator immobilization and ARGET ATRP of methyl acrylate from wood surfaces.

Prior to modification, the wood flour was extracted for 16 h with a Soxhlet apparatus using dichloromethane (DCM) as solvent and afterwards dried at 60 °C under reduced pressure. The modification with poly(methyl acrylate) (PMA) was performed in a two-step process, outlined in Scheme 1. First, the ATRP initiator was immobilized on the surface; second, polymer chains were grafted via surface-initiated ARGET ATRP. The immobilization was conducted through esterification of the surface hydroxyl groups with 2-bromoisobutyryl bromide (BIBB) in the presence of DCM as solvent and triethylamine (TEA) as auxiliary base. 

**Figure 1:**
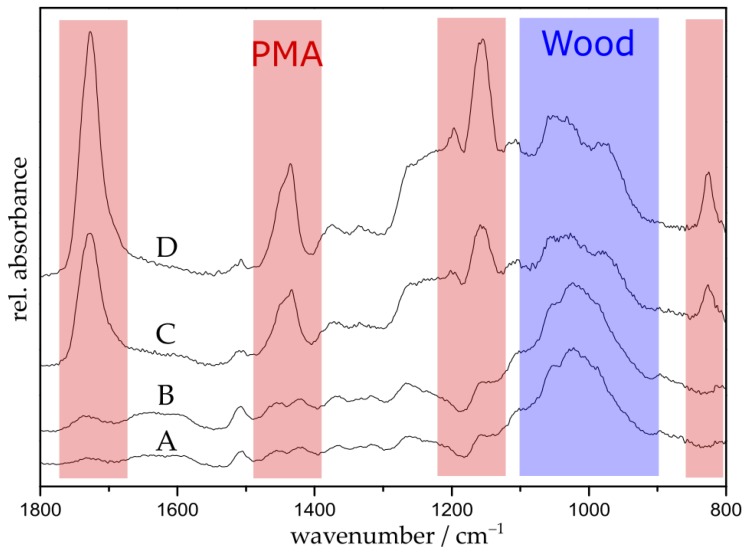
FTIR spectra of untreated wood (**A**); initiator-immobilized wood (**B**); and PMA-grafted wood with polymerization times of 2 h (**C**) and 5 h (**D**).

The grafting of poly(methyl acrylate) was performed by placing the initiator-functionalized particles in a one-pot mixture of methyl acrylate (MA), anisole, Copper(II) bromide (CuBr_2_), pentamethyldiethylenetriamine (PMDETA), and ascorbic acid (AsAc). The suspension was purged with argon for 5 min. The polymerization was conducted at 60 °C with polymerization times ranging between 45 min and 9.5 h. The choice of anisole as solvent is crucial because on the one hand, it is a good solvent for the catalyst system and on the other hand, it is a poor solvent for AsAc. Since AsAc is a very strong reducing agent, poor solubility limits its reduction potential, preventing high radical concentrations and, therefore, loss of control over the polymerization [42]. Additionally, the ligand PMDETA was used in a tenfold excess in order to ensure the formation of the intended metal ligand complex [43,44]. After the polymerization, the samples were washed with an excess of tetrahydrofuran. The grafted samples were characterized by FTIR spectroscopy, scanning electron microscopy (SEM), thermogravimetric analysis (TGA), and water contact angle (WCA) measurements. Figure 1 shows the recorded FTIR spectra of untreated wood (A), initiator-immobilized wood (B), and PMA-grafted wood with polymerization times of 2 h (C) and 5 h (D). For reason of simplicity, spectra of other polymerization times are exclusively shown in the supporting materials (Figure S1). It can be clearly seen that polymerization results in the emergence of four distinct peaks at 1725, 1435, 1153, and 826 cm^−1^ (shown in red) attributed to PMA [45]. The intensity of the PMA peaks increases whereas the intensity of the wood peaks at 1100–900 cm^−1^ (shown in blue) decreases with progressing polymerization time [46]. 

**Figure 2:**
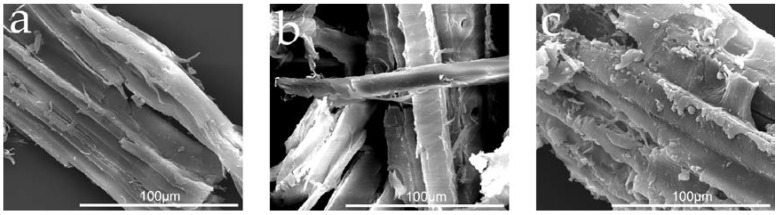
SEM images of untreated wood flour (**a**); initiator-functionalized wood flour (**b**); and PMA-grafted wood flour with a polymerization time of 5 h (**c**).

SEM studies were employed to capture the surface topography of unmodified wood (Figure 2a), initiator-functionalized wood (Figure 2b), and PMA-grafted wood particles after 5 h (Figure 2c). Before modification, wood exhibits a fibrous structure with a smooth surface. Immobilization of the ATRP initiator resulted in no significant topographical change. After graft polymerization, however, the surface was rough and irregular due to a clearly visible polymer layer [34].

**Figure 3:**
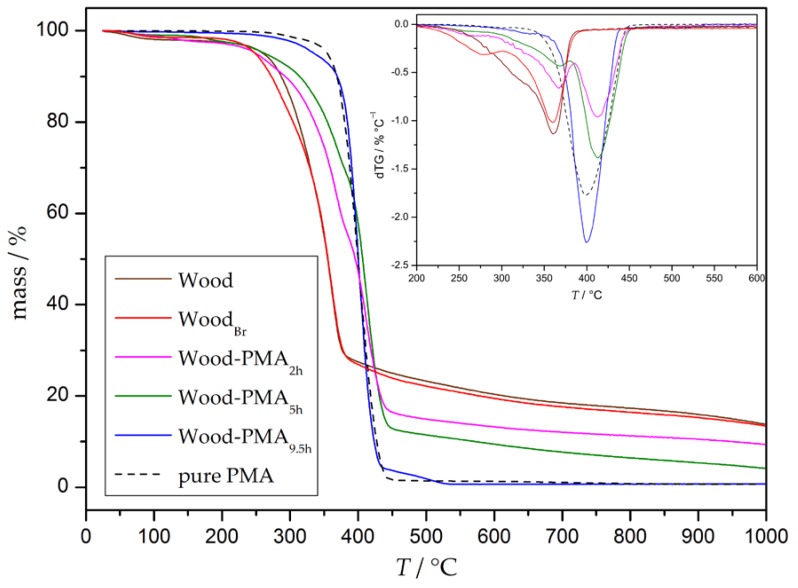
Thermograms and derivations of untreated wood, Wood_Br_, PMA-grafted wood samples, and pure PMA.

Thermogravimetric analysis was conducted to study the thermal behavior of the grafted samples. The thermogram (TG) and the respective derivation (DTG) of unmodified and modified wood as well as of pure PMA are depicted in Figure 3, and the corresponding decomposition temperatures (*T*_on_: onset degradation temperature, *T*_max_: temperature at the maximal degradation state) and mass losses (ML) are summarized in Table 1. Untreated wood starts to degrade sharply at 200 °C with a maximum at 360 °C and a total mass loss of 69% in this stage. Afterwards, at 450 °C, wood decomposes further at a much slower steady rate. This result is in good agreement with decomposition measurements of the three main wood components—cellulose, hemicellulose, and lignin. The degradation of cellulose and hemicellulose takes place at a high rate at 200–380 °C and 250–380 °C, whereas lignin slowly decomposes at 180–900 °C [47]. Initiator immobilization (Wood_Br_) results in a decrease of the onset temperature to 160 °C and a similar *T*_max_. Accordingly, it can be concluded that initiator immobilization on the wood surface decreases thermal stability, due to two reasons: first, esterification of cellulose damages its crystallinity; and second, hydrogen bromide may be formed, which catalyzes the decomposition of wood [31,33]. Pure PMA starts to decompose at 220 °C with a maximum decomposition rate at 400 °C and a total mass loss of 98%. Grafted samples (Wood-PMA_xh_) show a two-step degradation profile; the steps fluently merge into each other and can be assigned to a wood decomposition stage (*T*_max_ approx. 360 °C) and a PMA decomposition stage (*T*_max_ approx. 400 °C). In general, surface-initiated graft polymerization of PMA results in an increased thermal stability indicated by a decreased mass loss below 200 °C. A longer polymerization time leads to an increased *T*_on_ in the wood stage and, therefore, a shift to pure PMA. A closer look at the mass losses at both stages shows that the ML decreases in the wood stage with progressing polymerization time (Table 1, blue arrow), but increases in the PMA stage (Table 1, red arrow). 

In addition to the determination of the thermal behavior of the composites, TGA can be used to estimate the ratio of polymer grafted on the particles. A closer look at the decomposition of the pure polymer reveals that it is almost completely decomposed at 450 °C. Under the assumption that wood and PMA in the composites exhibit the same thermal behavior as the pure wood and pure PMA, the leftover mass should exclusively belong to the undecomposed part of wood at this temperature. The polymer content can be calculated with the following Equations (1)–(3), where a given *w* represents the mass fractions of the respective species.

(1) $$w_{\mathrm{polymer}}=1-w_{\mathrm{wood}}$$

(2) $$w_{450{}^{\circ}C}=0.24\;w_{\mathrm{wood}}$$

(3) $$w_{\mathrm{polymer}}=1-\frac{w_{450{}^{\circ}C}}{0.24}$$

In general, the mass fraction of grafted polymer (*w*_polymer_) in a composite corresponds to 1 minus the mass of wood (*w*_wood_), as can be seen in Equation (1). Untreated wood decomposes to a remaining mass of 24% at the temperature of 450 °C (as can be seen in Figure 3), which means that the mass fraction of the measured grafted wood (*w*_450°C_) can be expressed by Equation (2). Combination of Equations (1) and (2) results in the final relation of *w*_polymer_ and *w*_450°C_. The determined residual masses *w*_450°C_ at 450 °C and calculated polymer content *w*_polymer_ are shown in Table 2. As can be seen from the data, the residual mass of the grafted particles at 450 °C decreases with progressing polymerization time, which leads to higher amounts of grafted polymer according to Equation (3).

DSC measurements were conducted to complete the thermal characterization of the PMA-grafted wood (the spectra are depicted in the supporting materials, Figure S2). All samples showed a glass transition temperature (*T*_g_) of 19 ± 1 °C, which is similar to the *T*_g_ of the matrix polymer and became more pronounced with increasing polymerization time. As a result, utilization of these grafted particles as a filler in thermoplastic does not change the thermal behavior of the resulting composite and, therefore, its scope of application.

**Figure 4:**
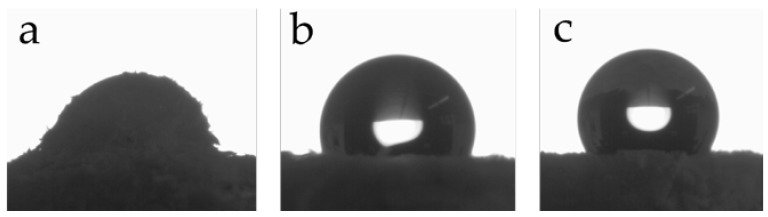
Water contact angle measurements of untreated wood (**a**) and PMA-grafted wood with polymerization times of 2 h (**b**) and 5 h (**c**).

Water contact angle measurements were performed to evaluate the hydrophobic character of the grafted particles. Prior to measurements, the flour was compressed to obtain a flat surface. Figure 4a–c show the contact angles for unmodified wood and PMA-grafted wood with polymerization times of 2 and 5 h. The unmodified wood surface is very hydrophilic due to numerous hydroxyl groups which results in an immediate absorption of the water and swelling of the sample. In contrast, grafting of PMA on the surface over 2 or 5 h leads to contact angles of 112° and 123°, respectively, which are maintained for several minutes and demonstrate the successful grafting of PMA.

### 3.2. Selective Cleavage of the Grafted Polymer under Mild Conditions

**Scheme 2:**
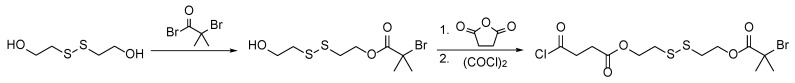
Synthesis of the disulfide-containing ATRP initiator for subsequent detachment of polymer grafts under mild conditions (according to literature [39]).

Usually, a sacrificial initiator is deployed to spectroscopically follow the progress of the polymerization and to obtain information about the control over the polymerization [12]. Free polymer formed from the sacrificial initiator can be analyzed by size-exclusion chromatography (SEC), which provides estimations of the molecular weight and dispersity of the polymer grafts [48,49]. Alternatively, polymer grafts can be cleaved off and analyzed directly, but this often requires harsh conditions and leads to degradation of the surface [50,51]. In this study, no sacrificial initiator was utilized, because the formation of free polymer was undesired. Instead, an ATRP initiator bearing a disulfide group was immobilized on the surface, allowing the detachment of the polymer grafts under mild conditions [52,53]. Subsequently, the detached polymer was analyzed by SEC to receive information about the molar mass and dispersity. The synthesis of the disulfide initiator is outlined in Scheme 2. 

The immobilization of this initiator was conducted comparably to the immobilization of BIBB except that pyridine (Py) was used as an auxiliary base. It is evident from the energy-dispersive X-ray spectroscopy (EDX) data given in Table 3 that the immobilization was successful as indicated by the resulting bromine content of 2.0%, which is very similar to the obtained bromine content of 1.7% with BIBB as initiator. It is known in the literature that the extent of polymerization (length of polymer chains and dispersity) is not greatly influenced by the total number of initiator sites on the substrate [50], which means that the grafting densities of BIBB-modified and disulfide-initiator-modified wood should be comparable. Afterwards, the disulfide-modified particles were grafted with methyl acrylate via ARGET ATRP for 2 or 5 h.

**Scheme 3:**
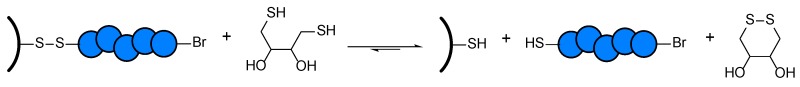
Schematic cleavage reaction of PMA-grafted wood containing a disulfide linker.

The resulting disulfide-containing particles were treated with a solution of 1,4-Dithiothreitol (DTT) and TEA in tetrahydrofuran (THF) at room temperature for several days to cleave off the grafted polymer. DTT is a reducing agent bearing two thiol groups which react selectively with the disulfide bond of the surface-tethered initiator [39,54]. In the reaction, the disulfide group of the substrate is reduced, leading to a surface-tethered thiol moiety and the release of the polymer into the solution with a thiol end group, as can be seen in Scheme 3. 

The polymer was analyzed by SEC and the obtained peak maximum of the number average molar mass (*M*_n,max_) and the dispersity (*Đ*) are displayed in Table 4. The data clearly show a sufficient control over the polymerization given by an increasing molar mass with progressing polymerization time and similar dispersities.

**Figure 5:**
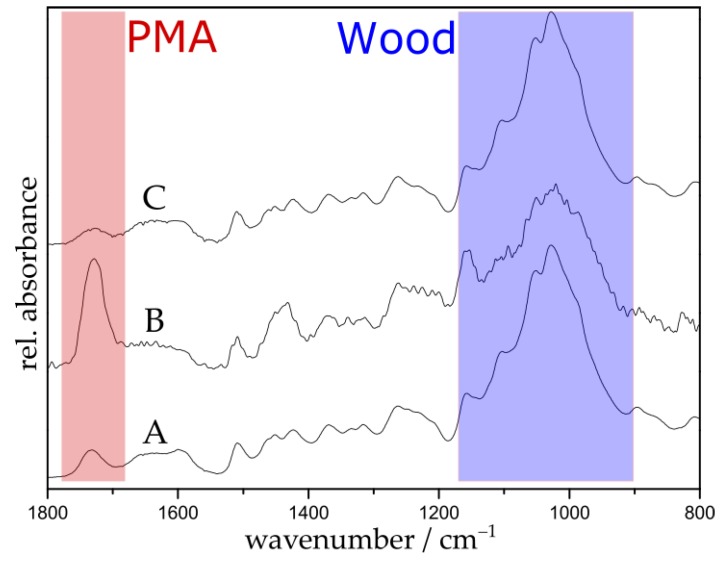
FTIR spectra of disulfide initiator-immobilized wood (**A**); PMA-grafted wood containing a disulfide linker with a polymerization time of 5 h (**B**); and DTT-cleaved wood (**C**).

ATR-FTIR spectroscopy was utilized to confirm the complete removal of the surface-tethered polymer with DTT. Figure 5 shows the spectra of the disulfide initiator-immobilized wood (A), PMA-grafted wood containing disulfide linker (B), and DTT-cleaved wood substrate (C). After cleavage, the polymer signal at 1725 cm^−1^ (red region) is substantially decreased and the resulting spectrum looks very similar to the spectrum of untreated wood (see Figure 1a); this confirms the successful cleavage of the polymer.

**Figure 6:**
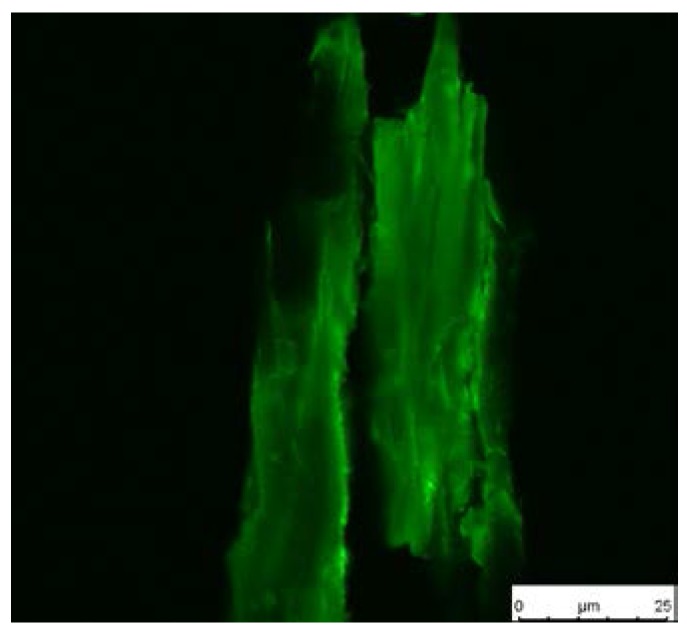
Fluorescence image of stained wood flour.

The thiol functionalities on the surface, which result from the reduction of the disulfide bonds, were made visible by staining with a fluorescence dye and were subsequently observed with confocal microscopy. The dye bears a maleimide moiety which readily reacts with thiol groups via a thiol-ene reaction. After immobilization of the dye, the sample was excited at 488 nm and detected at 500–570 nm. A green color shows the presence of former thiol groups and the intensity of the color is correlated with the initiator density. Figure 6 shows a dye-treated wood particle after cleavage of the polymer. The image reveals a satisfying surface coverage of the wood particle with a few regions with significantly higher thiol concentration. Untreated wood, which was used in a control experiment, showed no fluorescence after treatment with the dye (image not shown).

### 3.3. Preparation and Properties of Wood-Reinforced Thermoplastics

**Figure 7:**
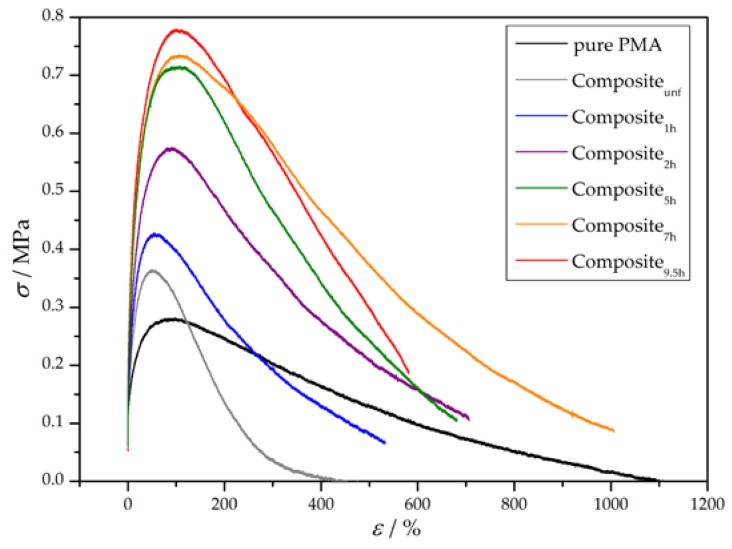
Representative stress-strain curves of pure PMA and wood-reinforced thermoplastics. Wood flour (5 wt %) with varying amounts of grafted polymer was incorporated into the matrix.

PMA-grafted wood flour was incorporated in a PMA matrix and its influence on the mechanical properties of the composites was investigated using uniaxial tensile testing. The matrix polymer PMA was synthesized via reversible addition-fragmentation (RAFT) polymerization. Dogbone specimens were prepared using solvent casting. Tensile testing was performed at room temperature with a constant strain of 0.25 mm·s^−1^. A constant weight percent of wood flour with a varying amount of grafted polymer was incorporated in the matrix and the influence on the Young’s modulus *E*, the yield strength σ_y_, and the toughness *U*_T_ was recorded. Samples of pure PMA and composites of unfunctionalized wood flour in PMA served as references to evaluate the reinforcing character of the grafted wood flour. Figure 7 shows typical stress-strain plots, chosen from a data set of three measurements per sample. The corresponding mechanical characteristics are summarized in Table 5. 

Pure PMA exhibits the typical behavior of a thermoplastic above its glass transition temperature. After a small increase of stress in the initial elastic region, the sample starts to flow due to a lack of internal cohesion until an elongation of about 1100% [55]. Incorporation of unfunctionalized wood flour (Composite_unf_) resulted in a similar Young’s modulus and a slightly increased yield strength, but the more noticeable change was a halving of the toughness. An increase in yield strength can be explained by the inherently stiff character of wood flour which leads to a higher force required. The dramatic drop in toughness is due to two reasons: first, incorporated stiff particles may disrupt the ability of unbranched polymer chains to flow past each other; second, unfunctionalized wood particles possess a highly hydrophilic surface, which results in poor interfacial adhesion with the hydrophobic polymer matrix and, therefore, an insufficient stress transfer between both phases [2,7]. In contrast, all composites made of grafted wood flour exhibit overall enhanced mechanical properties over pure PMA and the composite made with unfunctionalized wood flour. In general, the longer the polymerization time, the higher the reinforcing effect. The Young’s modulus increases almost linearly with the polymerization time, whereas the yield strength seems to start off linear as well but reaches saturation. However, the toughness—for which the area under the curve is a measure—seems to increase rapidly to a maximum at a polymerization time of between 5 and 7 h and decrease afterwards. It is important to note that the reinforcing character cannot purely be explained by a better interfacial adhesion mediated by longer polymer chains on the particle surface. Additionally, the intrinsic composition of the particles plays an important role. From a practical point of view, addition of the same weight fraction of grafted particles regardless of the polymerization time is a more natural approach compared with addition of a constant mass fraction of wood. Nevertheless, longer polymerization times result in lower wood-to-polymer ratios of the particles, as can be seen in Table 2. Based on the assumption that the discovered reinforcing effect exclusively results from the inherent mechanical properties of wood and the grafted polymer has no significant effect on the composite, a lower wood-to-polymer ratio should lead to weakened mechanical properties. Therefore, this effect counteracts the increased interfacial adhesion of grafted particles. However, tensile tests clearly showed that longer polymerization times are beneficial for the mechanical properties, probably due to an increased ratio of surface-bound polymer molar mass to matrix polymer molar mass. Consequently, this effect, in combination with the improved compatibility between wood and the polymer matrix, plays the major role in the reinforcing character. 

**Figure 8:**
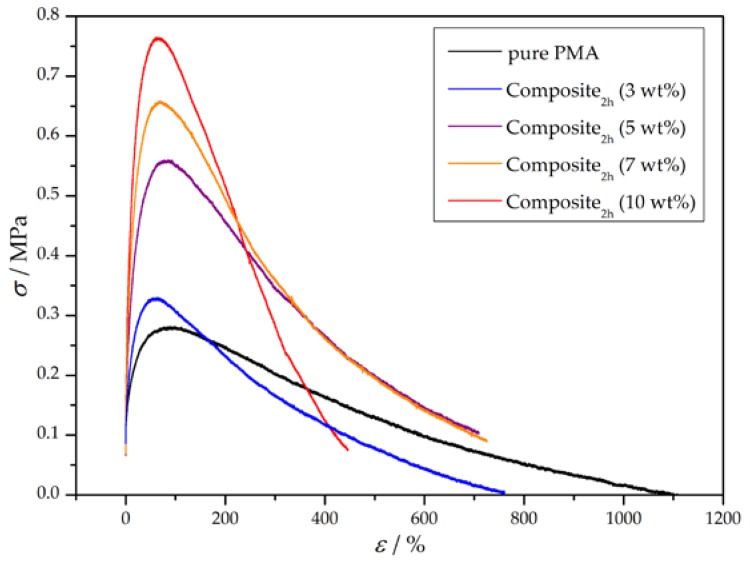
Representative stress-strain curves of pure PMA and wood-reinforced thermoplastics with a constant amount of grafted polymer and varying weight percentages.

Furthermore, wood particles with a constant amount of grafted polymer were incorporated in the matrix in different mass percentages. For this purpose, wood particles with a polymerization time of 2 h were added in 3, 5, 7, and 10 wt % into a PMA matrix and the mechanical properties of the composites were determined. Based on the preparation method of the composite, addition of 10 wt % grafted particles was the maximum amount which could be added, because otherwise the dogbone specimen exhibited a very heterogenous structure. The resulting stress–strain curves are depicted in Figure 8 and the corresponding mechanical characteristics are summarized in Table 6. Generally, the higher the added amount of grafted wood particles, the higher the Young’s modulus and tensile strength, whereas the toughness of the composite reaches a maximum at around 5 to 7 wt %. The Young’s modulus and yield strength are not significantly affected after addition of 3 wt % grafted particles, but start to rapidly increase with higher addition rates and seem to reach a saturation afterwards. As a result, addition of 7 wt % seems to be the optimum regarding the examined mechanical characteristics.

## 4. Conclusions

The modification of wood flour was accomplished via surface-initiated ARGET ATRP of methyl acrylate with an environmentally friendly reducing agent (AsAc) in the absence of a sacrificial initiator. The amount of grafted polymer on the wood could be estimated via TGA measurements and control over the polymerization (polymer length and dispersity) was verified via detachment of the polymer under mild conditions. Additionally, wood-flour-reinforced thermoplastics with PMA as matrix were produced and investigated by means of tensile testing. Addition of unfunctionalized wood flour in PMA results in worse mechanical properties than pure PMA, whereas addition of grafted wood flour leads to increased Young’s modulus, yield strength, and toughness. The findings herein presented may also be extended to other polymer-wood composites.

## Figures and Tables

**Table 1:** Thermal degradation temperatures and mass losses (MLs) of untreated wood, Wood_Br_, PMA-grafted samples, and pure PMA.

	Wood Stage			PMA Stage	
	*T*_on_/°C	*T*_max_/°C	ML/%	*T*_max_/°C	ML/%
Wood	200	360	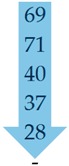	-	-
Wood_Br_	160	360		-	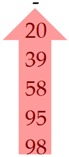
Wood-PMA_0.75h_	160	360		410	
Wood-PMA_2h_	170	365		410	
Wood-PMA_5h_	190	370		410	
Wood-PMA_9.5h_	190	-		400	
PMA	220	-	-	400	

**Table 2:** Estimation of the amount of grafted PMA for different polymerization times.

	*w*_450°C_/%	*w*_polymer_/%
Wood-PMA_0.75h_	24	0
Wood-PMA_2h_	16	33
Wood-PMA_5h_	13	46
Wood-PMA_9.5h_	4	83

**Table 3:** Comparison of both immobilized ATRP initiators.

ATRP initiator	Base	Br wt % (EDX)
BIBB	TEA	1.7 ± 0.7
Disulfide	Py	2.0 ± 0.6

**Table 4:** Properties of cleaved polymer grafts.

*t*/h	*M*_n,max_/10^3^ g·mol^−1^	*Đ*
2	290	1.84
5	410	1.88

**Table 5:** Mechanical characteristics of pure PMA and wood-reinforced thermoplastics. Wood flour (5 wt %) with varying amounts of grafted polymer was incorporated into the matrix.

Sample	*E*/MPa	σ_y_/MPa	*U*_T_/MPa
pure PMA	8 ± 3	0.27 ± 0.06	126 ± 31
Composite_unf_	7 ± 3	0.30 ± 0.17	50 ± 17
Composite_1h_	11 ± 1	0.40 ± 0.03	106 ± 18
Composite_2h_	20 ± 2	0.58 ± 0.02	235 ± 16
Composite_5h_	26 ± 1	0.73 ± 0.02	297 ± 5
Composite_7h_	29 ± 2	0.74 ± 0.01	398 ± 15
Composite_9.5h_	36 ± 2	0.79 ± 0.02	299 ± 68

**Table 6:** Mechanical characteristics of pure PMA and wood-reinforced thermoplastics with a constant amount of grafted polymer and varying weight percentages.

Sample	*E*/MPa	σ_y_/MPa	*U*_T_/MPa
pure PMA	8 ± 3	0.27 ± 0.06	126 ± 31
Composite_2h_ (3 wt %)	8 ± 1	0.32 ± 0.01	97 ± 13
Composite_2h_ (5 wt %)	20 ± 2	0.58 ± 0.02	235 ± 16
Composite_2h_ (7 wt %)	22 ± 3	0.65 ± 0.01	228 ± 34
Composite_2h_ (10 wt %)	22 ± 8	0.74 ± 0.14	166 ± 49

## Supplementary Materials

The following are available online at http://www.mdpi.com/2073-4360/10/4/354/s1. Figure S1: FTIR spectra of untreated wood (**A**), initiator-immobilized wood (**B**), and PMA-grafted wood with polymerization times of 45 min (**C**), 2 h (**D**), 5 h (**E**), and 9.5 h (**F**). Figure S2: DSC curves of PMA-grafted wood and pure PMA. All measured samples showed a glass transition temperature of 19 ± 1 °C.
