# Supplementary material for: Surface Modification of Wood Flour via ARGET ATRP and Its Application as Filler in Thermoplastics
Source: Polymers (Basel). 2018 Mar 22;10(4):354. doi: 10.3390/polym10040354 (PMC6415022; doi:10.3390/polym10040354)
Supplement: Supplementary file 1 [file polymers-10-00354-s001.pdf]

polymers

MDPI

Article

Surface Modification of Wood Flour via ARGET ATRP and Its Application as Filler in Thermoplastics

Martin Kaßel, Julia Gerke, Adrian Ley and Philipp Vana*

Institute of Physical Chemistry, Georg-August-University Göttingen, Tammannstr. 6, D-37077 Göttingen, Germany

* Correspondence: pvana@uni-goettingen.de; Tel.: +49-(0)551-39-12753

Supporting Information:

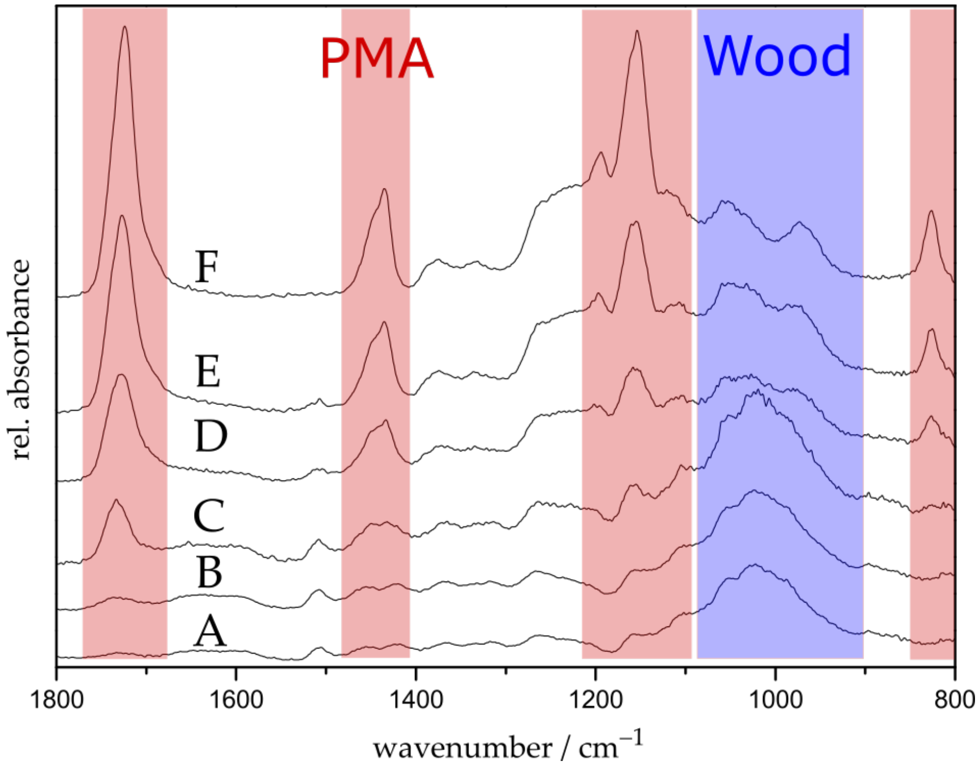

Figure S1: FTIR spectra of untreated wood (**A**), initiator-immobilized wood (**B**) and PMA-grafted wood with polymerization times of 45 min (**C**), 2 h (**D**), 5 h (**E**) and 9.5 h (**F**).

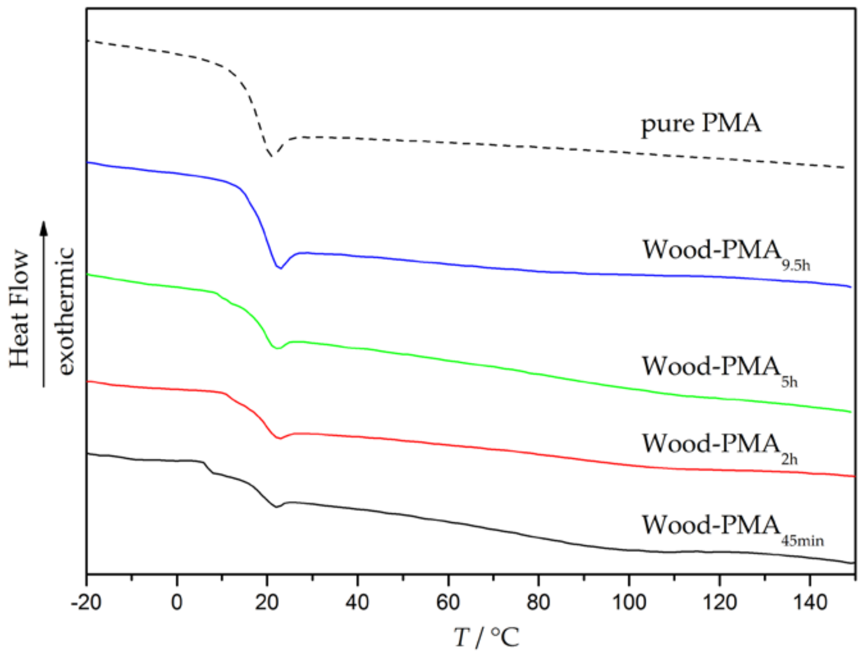

Figure S2: DSC curves of PMA-grafted wood and pure PMA. All measured samples showed a glass transition temperature of 19 ± 1 °C.
